# *Large1* gene transfer in older *myd* mice with severe muscular dystrophy restores muscle function and greatly improves survival

**DOI:** 10.1126/sciadv.abn0379

**Published:** 2022-05-25

**Authors:** Takahiro Yonekawa, Adam J. Rauckhorst, Sara El-Hattab, Marco A. Cuellar, David Venzke, Mary E. Anderson, Hidehiko Okuma, Alvin D. Pewa, Eric B. Taylor, Kevin P. Campbell

**Affiliations:** 1 Senator Paul D. Wellstone Muscular Dystrophy Specialized Research Center, Department of Molecular Physiology and Biophysics and Department of Neurology, Roy J. and Lucille A. Carver College of Medicine, Howard Hughes Medical Institute, The University of Iowa, Iowa City, IA 52242, USA.; 2 Department of Molecular Physiology and Biophysics, Fraternal Order of Eagles Diabetes Research Center (FOEDRC), and FOEDRC Metabolomics Core Facility, Roy J. and Lucille A. Carver College of Medicine, The University of Iowa, Iowa City, IA 52242, USA.

## Abstract

Muscular dystrophy is a progressive and ultimately lethal neuromuscular disease. Although gene editing and gene transfer hold great promise as therapies when administered before the onset of severe clinical symptoms, it is unclear whether these strategies can restore muscle function and improve survival in the late stages of muscular dystrophy. *Large^myd^/Large^myd^* (*myd*) mice lack expression of *like-acetylglucosaminyltransferase-1* (*Large1*) and exhibit severe muscle pathophysiology, impaired mobility, and a markedly reduced life span. Here, we show that systemic delivery of AAV2/9 CMV *Large1* (AAV*Large1*) in >34-week-old *myd* mice with advanced disease restores matriglycan expression on dystroglycan, attenuates skeletal muscle pathophysiology, improves motor and respiratory function, and normalizes systemic metabolism, which collectively and markedly extends survival. Our results in a mouse model of muscular dystrophy demonstrate that skeletal muscle function can be restored, illustrating its remarkable plasticity, and that survival can be greatly improved even after the onset of severe muscle pathophysiology.

## Teaser

*Large1* gene transfer improves muscle function and extends survival in older mice with severe muscular dystrophy.

## INTRODUCTION

Substantial progress has been made toward unraveling the genetic basis of various muscular dystrophies, yet existing therapies are limited and do not prevent the unchecked skeletal muscle degeneration that eventually leads to death. Gene therapy is an attractive approach for these individuals as it enables the delivery of a functional copy of a gene or repairs the mutated locus [for reviews, see (*1*–*5*)]. Recently, adeno-associated virus (AAV)-mediated gene transfer has shown great promise for treating patients with neuromuscular diseases, such as spinal muscular atrophy, when administered before severe clinical symptoms arise (*6*). Similarly, preclinical studies in which adenoviral or AAV-mediated gene transfer is administered at a young age before the onset of severe symptoms shows great promise in various mouse models of muscular dystrophy (*7*–*14*). Also, an established dystrophic state in aged *mdx* mice is improved upon AAV-mediated gene transfer (*15*). However, *mdx* mice do not exactly reproduce the severity of pathology exhibited by patients with Duchenne muscular dystrophy, except for in the diaphragm musculature, which limits the extrapolation of these results to human disease (*16*). Thus, the efficacy of AAV-mediated gene therapy in treating severe muscle degeneration that is associated with advanced stages of muscular dystrophy has not been fully elucidated.

Dystroglycan (DG) is a widely expressed high-affinity extracellular matrix (ECM) receptor that is highly glycosylated and involved in a variety of physiological processes (*17*–*20*). In skeletal muscle, DG is part of the dystrophin-glycoprotein complex, which establishes a continuous link between ECM proteins (laminin, agrin, and perlecan) and the cytoskeleton (*20*–*23*). Dystroglycanopathies are muscular dystrophies (*17*–*19*) characterized by an absence or reduction in matriglycan, a linear repeating disaccharide of alternating xylose and glucuronic acid that binds to the laminin G–like domains of ECM proteins with high affinity (*24*, *25*). Thus far, mutations in at least 18 human genes are known to disrupt the synthesis of matriglycan and cause dystroglycanopathy (*25*), including those in the *like-acetylglucosaminyltransferase-1* (*LARGE1*) gene (*26*–*28*), which encodes a xylosyl- and glucuronosyltransferase that modifies α-DG with matriglycan (*24*, *25*).

*Large^myd^/Large^myd^* (*myd)* mice are an excellent model for therapeutic studies of dystroglycanopathies because they lack matriglycan and exhibit severe skeletal muscle pathophysiology, impaired mobility, reduced body weight (BW), and a markedly reduced life span (only 50% of the *myd* mice survive to 35 weeks of age) (*17*, *29*–*31*). Using both noninvasive and invasive techniques, we performed a comprehensive study to test the ability of systemic AAV-mediated gene transfer of *Large1* to improve skeletal muscle function and survival in *myd* mice with advanced disease. We show that this treatment in older *myd* mice with severe muscular dystrophy attenuates skeletal muscle degeneration and improves motor and respiratory function as well as systemic metabolism, which collectively extends survival of *myd* mice. Thus, our study provides proof of concept that functional and pathological features of muscular dystrophy are treatable, even after severe muscle degeneration is established.

## RESULTS

### *Large^myd^/Large^myd^* (*myd*) mice show growth retardation, progressive muscle pathophysiology, and a shortened life span

To evaluate the therapeutic effect of treating established dystroglycanopathy, we targeted aged *myd* mice as they exhibited severe muscle pathophysiology. Before gene transfer studies, we analyzed the progression of disease in our *Large^myd^/Large^myd^* (*myd*) mouse colony on the C57BL/6 genetic background (92.5% C57BL/6) from the newborn period to 35 weeks of age. *myd* mice were generated by mating heterozygous +/*myd* mice and were significantly smaller than their littermates (+/+ or +/*myd*) at postnatal day 3, weighing 1.8 ± 0.3 g (mean ± SD, *n* = 18) versus 2.2 ± 0.5 g (*n* = 143). *myd* mice also showed consistently slower growth compared to littermate controls, although males and females continued to grow until 20 or 15 weeks of age, respectively, after which they exhibited a significant decline in BW (fig. S1, A and B). At 35 weeks of age, the weight of *myd* mice was markedly less than that of littermate controls: 21.4 ± 2.7 g (*myd*) versus 42.4 ± 6.1 g (littermate controls) in male and 16.7 ± 2.7 (*myd*) versus 34.2 ± 5.5 g (littermate controls) in female. The survival of *myd* mice was considerably shorter than that of littermate controls, with only 35% surviving to 40 weeks of age and no mice living past 60 weeks of age (fig. S1C). In addition, thoracic kyphosis and muscle wasting were evident at ∼40 weeks of age in *myd* mice (fig. S1D). Fibrous and adipose tissue infiltration was prominent in *myd* mice that were older than 35 weeks, with numerous small, round-shaped fibers observed upon histological analysis, indicative of end-stage pathology (fig. S2, A and B). Matriglycan-positive α-DG (functionally glycosylated) was localized in the muscle sarcolemma of C57BL/6 [wild-type (WT)] mice but absent in *myd* mice (fig. S2C).

### Systemic AAV-mediated gene transfer of *Large1* restores skeletal muscle function and improves survival in adult *myd* mice

**Fig. 1.**
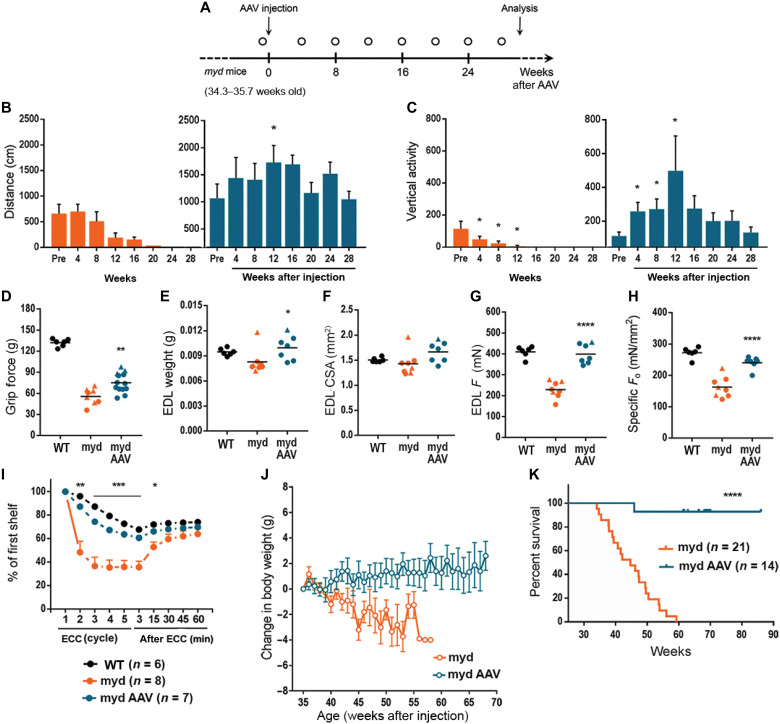
*Large1* gene transfer improves skeletal muscle function and extends survival. (**A**) Experimental outline. Mice were weighed weekly; open circle, locomotor activity determined. (**B** and **C**) Spontaneous locomotion (distance) and rearing (vertical activity). Untreated, orange; treated, blue. (**D** to **H**) Forelimb grip strength (D), EDL muscle weight (E), EDL muscle CSA (F), EDL isometric tetanic force (*F*_o_) (G), and specific isometric tetanic force (*F*_o_/CSA) (H) (black; C57BL/6J (WT)); treated *myd* (myd AAV), age >60 weeks; untreated *myd* (myd), age 46.5 ± 6.5 weeks. (**I**) Percentages of *F*_o_ in mice at indicated time after ECC relative to *F*_o_ at first ECC. (**J**) Changes in body weight relative to weight at 35 weeks of age. (**K**) Survival curves of mice in the indicated treated groups. Symbols, individual mice; bars, means ± SEM. C57, black; myd, orange; myd AAV, blue.

We next performed systemic AAV-mediated gene transfer of *Large1* in adult *myd* mice at an age when approximately 50% survived, and assessed the effects on muscle function and survival. These mice exhibit severe skeletal muscle pathophysiology, and since both DG and *Large1* are widely expressed in many tissues, we used systemic AAV gene transfer to obtain extensive and widespread expression of *Large1* in all muscles. We randomly assigned 35 *myd* mice that were 34 weeks or older to untreated or treated groups. Twenty-one mice were left untreated, whereas 14 mice were injected once with AAV*Large1* at age 38.4 ± 2.8 weeks (mean ± SD). A series of noninvasive and invasive therapeutic readouts were compared between the two groups, including BW, survival, muscle and respiratory function, muscle physiology, histological evaluation, plasma metabolomics, and biochemical analysis of α-DG. Mice were weighed weekly and locomotor activity was assessed every 4 weeks (see Fig. 1A for experimental outline). BW and locomotor activity were compared between baseline (i.e., at enrollment for untreated group or before AAV*Large1* injection for treated group) and each assessment time point. Respiratory function was assessed before untreated mice were euthanized or when AAV-treated mice reached 60 weeks of age. Muscle physiology was evaluated immediately before euthanasia, and tissues were subsequently harvested for further analysis.

Untreated *myd* mice (34 to 35 weeks old) showed a marked reduction in locomotor activity, as measured by distance traveled, or rearing behavior, as measured by vertical activity, and displayed progressive deterioration in motor performance until euthanasia was necessary (Fig. 1, B and C) compared to 37-week-old WT mice (fig. S3). In contrast, AAV-mediated gene transfer of *Large1* in *myd* mice restored locomotor and rearing activities, and mice were able to maintain motor performance until they were euthanized for analysis at age 69.7 ± 5.1 weeks (Fig. 1, B and C).

Forelimb grip strength was also markedly reduced in 46.5-week-old *myd* mice relative to WT mice aged 61.1 weeks (Fig. 1D). The muscle weight and cross-sectional area (CSA) of dissected extensor digitorum longus (EDL) muscles were smaller (Fig. 1, E and F), and the absolute isometric tetanic force (*F*_o_) and the size-normalized isometric tetanic force (*F*_o_/CSA) were markedly lower in *myd* mice compared to those of WT mice (Fig. 1, G and H), suggesting that the muscles were not only atrophic but also highly degenerated. In contrast, AAV*Large1-*treated *myd* mice showed a significant increase in forelimb grip strength (Fig. 1D); however, the muscle weight and CSA of isolated EDL muscles only increased modestly (Fig. 1, E and F).

Systemic transduction of *myd* mice with AAV*Large1* of *myd* mice also resulted in a significant increase in *F*_o_ and *F*_o_/CSA of EDL muscles compared to untreated mice (Fig. 1, G and H), demonstrating that even at advanced stages of disease muscle contractile properties can be improved by delivering a functional copy of *Large1*. In addition, EDL muscles in untreated mice were highly susceptible to injury induced by eccentric contractions (ECCs), and tetanic *F*_o_ force dropped by 63% after the third ECC (Fig. 1I). In contrast, EDL muscles in AAV*Large1-*treated mice tolerated a sequence of five ECCs, although the force drops induced by the ECC protocol were slightly greater than those in WT muscles (Fig. 1I). It should be noted that muscle size, contractile properties, and lengthening contraction-induced muscle damage are similar in EDL muscles from +/+ or +/*myd* mice (fig. S4).

At 35 weeks of age (just before AAV treatment), there was no significant difference in BWs between *myd* mice that were untreated or treated with AAV*Large1* (fig. S5A). However, after 35 weeks, untreated *myd* mice displayed a significant decline in BW that progressed until euthanasia was required (Fig. 1J), whereas systemic gene transfer of *Large1* resulted in a mild gain in BW (Fig. 1J). All surviving *myd* mice older than 35 weeks of age were very thin, suggesting they had already developed severe muscle atrophy and degeneration, and they continued to decline without treatment (fig. S5B, left). The sizes of the gastrocnemius and quadriceps femoris muscles were markedly smaller in *myd* mice compared to those of WT mice. Nevertheless, AAV-mediated gene transfer of *Large1* resulted in mild growth of these muscles (fig. S5, C to F), although treated mice still looked thin (fig. S5B, right), suggesting that these muscles may be too severely affected at later stages of disease to recover to normal weights. Notably, AAV-mediated gene transfer significantly extended the survival of *myd* mice, as all but one mouse injected with AAV*Large1* lived to >65 weeks of age, whereas only 50% of the *myd* mice survived to 45 weeks of age (Fig. 1K).

### AAV*Large1* restores matriglycan expression on α-DG and improves muscle pathology

**Fig. 2.**
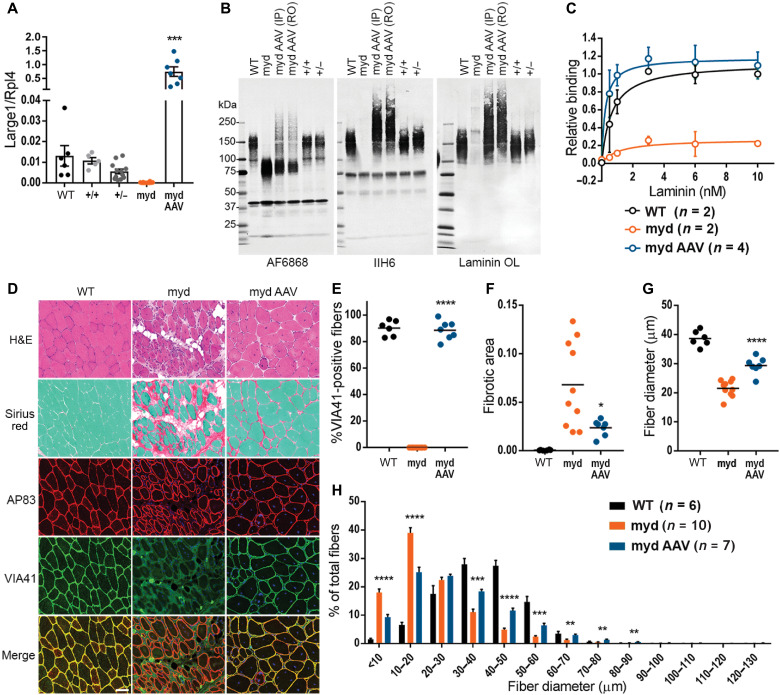
AAV*Large1* restores matriglycan on α-DG and laminin binding in skeletal muscles, which alleviates muscle pathology in *myd* mice. (**A**) *Large1* expression. (**B**) Western blot of core α-DG and β-DG (AF6868), matriglycan-positive α-DG (IIH6), or laminin (Laminin OL) in (A). (**C**) Solid-phase binding assay. (**D**) Representative cryosections stained with hematoxylin and eosin (H&E) or Sirius red and Fast Green, or used for immunofluorescence: β-DG (AP83); matriglycan-positive α-DG (VIA41). Scale bar, 50 μm. (**E** to **H**) Quantitative analysis of sections in (D). (E) VIA41-positive fibers. (F) Connective tissue deposition. (G) Average Feret’s diameter of fibers. (H) Percentage of fibers of indicated diameter. Symbols, individual mice; bars, means ± SEM. WT, C57Bl/6J; +/+, *Large^+/+^*; +/−, *Large^+/−^*; myd, untreated *myd*; myd AAV, AAV*Large1*-injected *myd* mice; IP, intraperitoneal; RO, retro-orbital.

As expected, *myd* muscles did not express *Large1* and the level of *Large1* expression in +/*myd* muscles was approximately half that observed in muscles from WT or +/+ mice (Fig. 2A). Western blot analysis of wheat germ agglutinin (WGA)–enriched *myd* muscle homogenates demonstrated that the transmembrane subunit of DG, β-DG, was normally expressed, whereas functional α-DG was absent in *myd* muscles, as assessed by reactivity to the IIH6 antibody (anti-matriglycan) or the laminin overlay assay (Fig. 2B). In addition, α-DG ligand binding was severely affected in *myd* muscles compared to WT muscles (Fig. 2C) corresponding to the fact that *myd* EDL muscles were highly susceptible to lengthening contractions (Fig. 1I). Of note, the quadriceps and EDL muscles in +/*myd* mice exhibited no difference in biochemical and physiological features, respectively, when compared to WT or +/+ muscles (Fig. 2B and fig. S4). The level of *Large1* expression in +/*myd* (+/−) muscles was around half of that of WT or +/+ levels (Fig. 2A), and +/*myd* muscles showed no biochemical or physiological abnormalities (Fig. 2B and fig. S4).

AAV-mediated gene transfer resulted in overexpression of *Large1* in *myd* mice, leading to hyperglycosylation of α-DG in the muscle sarcolemma (Fig. 2, A and B), as previously observed with adenovirus expressing *Large1* (*12*). Western blotting of WGA-enriched muscle homogenates after AAV-mediated systemic transduction showed that α-DG was highly glycosylated with matriglycan and bound laminin (Fig. 2B). Furthermore, restoring *Large1* expression in *myd* mice rescued α-DG ligand binding (Fig. 2C), explaining the observation that EDL muscles were protected from ECC-induced damage.

The improved phenotype in muscle size and function observed in *myd* mice upon AAV-mediated gene transfer of *Large1* prompted us to determine whether this treatment also improved skeletal muscle pathology. As expected, histological analysis of skeletal muscle in untreated *myd* mice showed marked variation in fiber size, numerous fibers with central nuclei, adipose tissue infiltration, and fibrosis (Fig. 2D). Immunofluorescence analysis also showed a lack of matriglycan on α-DG in untreated *myd* mice (Fig. 2D). In contrast, quantification of matriglycan-positive α-DG fibers revealed that almost all fibers expressed functional α-DG in *myd* mice after injection with AAV*Large1* (Fig. 2E). Furthermore, progression of fibrous tissue deposition was halted in treated mice when compared to untreated mice (Fig. 2F). In the absence of *Large1* expression, mice exhibited muscle wasting (fig. S5, C to F), associated with muscle fiber atrophy as revealed by an overall decrease in fiber diameter in untreated *myd* quadriceps femoris muscles (Fig. 2G), with a higher proportion of fiber diameters of 10 to 20 μm (Fig. 2H). In contrast, AAV-mediated overexpression of *Large1* increased overall muscle fiber diameter, as demonstrated by a greater percentage of fibers having a larger fiber diameter (Fig. 2, G and H).

### Effects of systemic AAV-mediated gene transfer of *Large1* on the diaphragm, cardiac muscles, and central nervous system

**Fig. 3.**
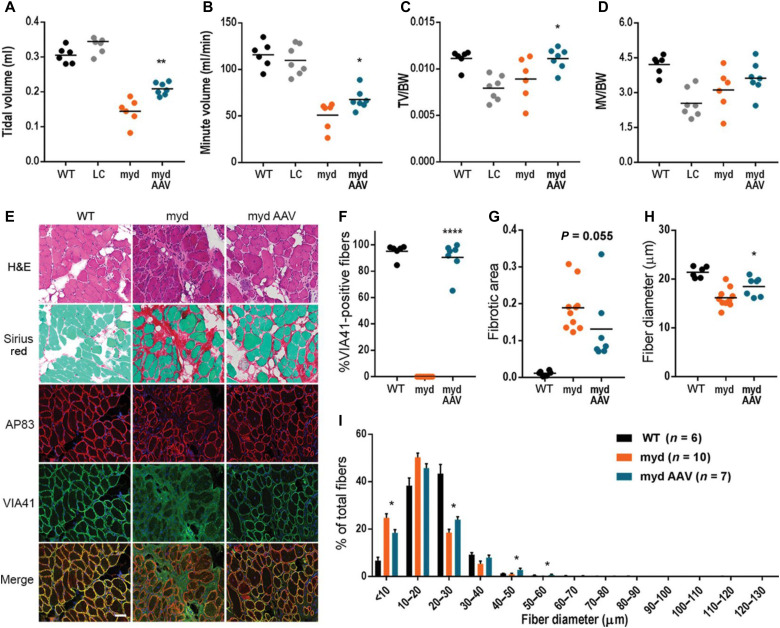
*Large1* gene transfer improves respiratory function and diaphragm pathology. Whole-body plethysmography for (**A**) TV, (**B**) MV, (**C**) TV normalized to body weight (TV/BW), and (**D**) MV normalized to weight (MV/BW). (**E**) Representative cryosections. Sections stained with H&E or Sirius red and Fast Green, or used for immunofluorescence: β-DG (AP83) and matriglycan-positive α-DG (VIA41). Scale bar, 50 μm. (**F** to **I**) Quantitative analysis of sections in (E). (F) VIA41-positive fibers. (G) Connective tissue deposition. (H) Average Feret’s diameter of fibers. (I) Percentage of fibers of indicated diameter. Symbols, individual mice; bars, means ± SEM. WT, C57BL/6J; myd, untreated *myd*; myd AAV, AAV*Large1*-injected *myd* mice; LC, littermate control (*Large^+/+^* or *Large*^+/-^).

Respiratory function is impaired in *myd* mice (*29*–*31*), as demonstrated by a marked reduction in tidal volume (TV) and minute volume (MV) compared to WT mice or littermate controls (Fig. 3, A and B). Moreover, *myd* mice displayed lower TV and MV normalized to BW (TV/BW and MV/BW) than WT mice or littermate controls (Fig. 3, C and D). A respiratory function test revealed that *myd* mice treated with AAV*Large1* had a higher TV and MV than untreated mice (Fig. 3, A and B), indicating that treated mice exhibit reduced forced breathing compared to untreated mice. In addition, TV/BW was moderately increased in treated mice versus untreated mice, whereas MV/BW was unaffected (Fig. 3, C and D), demonstrating that AAV-mediated overexpression of *Large1* ameliorated defects in respiratory muscle function.

We next investigated whether systemic gene transfer improves diaphragm pathology in *myd* mice. In untreated mice, fibrous tissue infiltration was prominent, and no muscle fibers expressed matriglycan-positive α-DG (Fig. 3, E and F). In contrast, treatment with AAV*Large1* restored expression of matriglycan on α-DG to similar levels observed in WT mice (Fig. 3F). Progression of connective tissue deposition was also prevented by AAV-mediated gene transfer of *Large1* relative to untreated mice (Fig. 3G). In addition, the overall fiber diameter of diaphragm muscles was increased (Fig. 3H), and an increased percentage of muscle fibers had a larger fiber diameter upon overexpression of *Large1* (Fig. 3I). Thus, AAV-mediated gene transfer of *Large1* caused muscle fibers in the diaphragm to increase in diameter, thus improving respiratory function.

We further investigated whether systemic gene delivery using AAV restores matriglycan on α-DG in cardiomyocytes in *myd* mice. No obvious pathology was observed in the absence of *Large1* (fig. S6A). Immunofluorescence analysis revealed that expression of matriglycan on α-DG was restored in hearts of *myd* mice treated with AAV*Large1* and was likely due to restored expression of the *Large1* gene (fig. S6, A and B). Of note, the subendocardial portion was less transduced (fig. S6A), indicating that AAV transduction is less efficient in this region. No difference in connective tissue infiltration was observed between untreated and treated mice, although two mice that were treated with AAV*Large1* had focal fibrous tissue deposition (fig. S6C). Therefore, AAV-mediated gene delivery of *Large1* restores glycosylated α-DG in cardiomyocytes, although the effects of this on cardiomyocyte function are difficult to discern in the *myd* mouse given the lack of phenotype in the heart in our *myd* mouse colony.

As in skeletal muscle, *Large1* was not expressed in the brain of *myd* mice, whereas gene expression levels in the brain of +/*myd* mice were around one-half of WT levels (fig. S7A). In addition, matriglycan-positive α-DG (functionally glycosylated) was similarly expressed in WT, +/+, and +/*myd* mice but absent in *myd* mice (fig. S7B). Retro-orbital (RO) or intraperitoneal AAV*Large1* injection resulted in a small amount of *Large1* expression in the brain of *myd* mice but not enough to restore matriglycan on α-DG or α-DG ligand binding (fig. S7, A and B). Considering that the brain of *myd* pups was well transduced with intravenous AAV*Large1*, poor penetration of adult *myd* brain remains to be resolved.

### Efficacy of AAV*Large1* gene transfer in normalizing whole-body metabolism

**Fig. 4.**
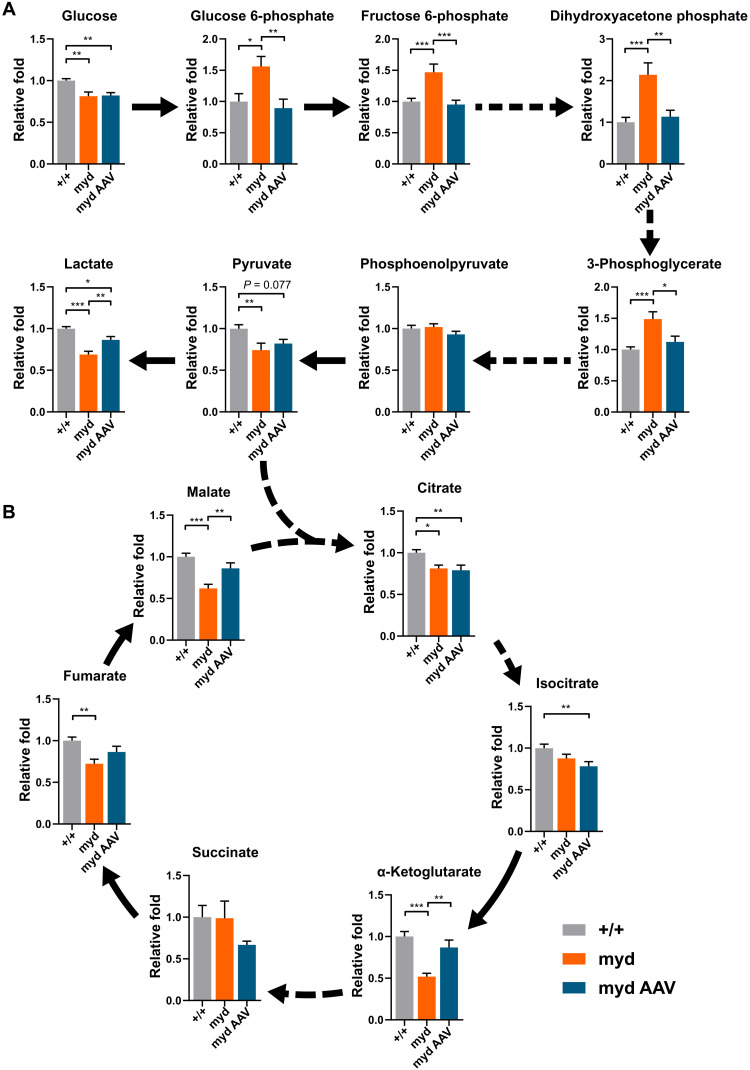
*Large1* is required for normal glycolytic and TCA cycle plasma profiles. Relative fold change of plasma: (**A**) glycolytic and (**B**) intermediate TCA cycle metabolites from *Large^+/+^* (+/+; gray bars, *n* = 11), untreated *myd* (myd; orange bars, *n* = 7), and *myd* mice treated with AAV*Large1* (myd AAV; blue bars, *n* = 6); solid and dashed arrows represent single and multiple metabolic reactions, respectively; *P < 0.05, **P < 0.01, and ***P < 0.001. Analysis by ordinary one-way analysis of variance (ANOVA) followed by post hoc Tukey’s multiple comparison test. Data presented as means ± SEM.

Skeletal muscle metabolism is a critical regulator of systemic metabolism that becomes abnormal in muscular dystrophies (*32*–*35*). The plasma metabolome is a useful readout of whole-body metabolism that reflects changes in muscle metabolism resulting from both altered basal nutrient utilization and locomotive activity. To determine the efficacy of AAV*Large1* gene transfer in normalizing whole-body metabolism, we performed targeted metabolomic profiling on plasma from *myd* mice with advanced disease. The plasma metabolome was abnormal in *myd* mice, with significant changes observed in 21 of 78 measured plasma metabolites, 10 of which were normalized upon AAV*Large1* gene transfer (table S1). Notably, glycolytic (Fig. 4A) and tricarboxylic acid (TCA) cycle (Fig. 4B) intermediates were enriched among altered metabolites, especially those restored by AAV*Large1*. *myd* mice exhibited increased early and mid-glycolytic intermediates and decreased lactate, suggesting altered coupling of early and late glycolysis (Fig. 4A). In parallel, TCA cycle intermediates malate, fumarate, and α-ketoglutarate were decreased, consistent with decreased TCA cycle amino acid influx through α-ketoglutarate in the absence of *Large1* (Fig. 4B). Overall, the metabolomic profiling of plasma showed that systemic metabolism was normalized following treatment with AAV*Large1*.

## DISCUSSION

Current therapies available to patients with muscular dystrophy are limited and neither restore normal skeletal muscle function nor improve survival. Gene therapy is an exciting therapeutic approach for the treatment of muscular dystrophy as it enables the delivery of a functional copy of a gene or repairs the mutated locus. Preclinical studies using adenoviral or AAV-mediated gene transfer to treat various mouse models of muscular dystrophy at a young age have been very successful in preventing skeletal muscle pathophysiology (*7*–*14*). However, the use of gene transfer has been difficult to study when applied as a treatment in the late stages, after the onset of severe muscle pathophysiology. Yet, these “restoration” studies are particularly important since many patients with muscular dystrophy have skeletal muscle degeneration before the onset of clinical symptoms and are often not diagnosed or treated until after these symptoms arise. Thus, we seek to understand the ability of gene therapy to repair severely damaged skeletal muscle and improve survival.

Our previous work with *Large1* gene transfer using adenovirus required direct muscle injections into young *myd* mice and showed localized prevention of dystrophic pathology (*11*). In addition, *Large1* gene transfer into healthy muscle did not cause abnormalities, although *Large1* was highly overexpressed, and α-DG was hyperglycosylated (*11*). Transgenic rescue of *myd* mice also had no overt ill effect, which induced a 100-fold overexpression of LARGE (*36*). This same overexpression in mice on a WT background was reported to be similarly well tolerated (*37*). Here, we demonstrated that AAV-mediated gene transfer induced a 100-fold overexpression of *Large1* beyond physiological levels, and that hyperglycosylated α-DG improved the link of DG to the basement membrane in *myd* muscles with no adverse effects.

To test whether severely damaged skeletal muscle can be repaired and overall life span can be improved, we performed a comprehensive study using systemic AAV-mediated gene transfer of *Large1* in *myd* mice with advanced disease. Since α-DG and *Large1* are widely expressed, we used the cytomegalovirus (CMV) promoter to drive *Large1* expression and ensure that α-DG glycosylation was restored. To evaluate the effectiveness of AAV-mediated gene transfer of *Large1*, we used various therapeutic readouts, including BW, survival, muscle and respiratory function, muscle physiology, histological evaluation, plasma metabolomics, and biochemical analysis of α-DG.

We first used noninvasive techniques to monitor the effectiveness of systemic treatment with AAV*Large1* to restore muscle function in older *myd* mice. Both locomotor and rearing activities were restored, and mice were able to maintain motor performance. AAV*Large1-*treated *myd* mice also showed a significant increase in forelimb grip strength. Moreover, AAV-mediated expression of *Large1* ameliorated defects in respiratory muscle function and normalized systemic metabolism, as determined by metabolomic profiling on plasma. Although the precise mechanisms underlying these metabolomic changes must still be determined, these results identify plasma metabolic signatures that are associated with the absence of *Large1* in *myd* mice, and that are restored upon AAV*Large1* gene transfer. Consequently, future studies should avail themselves to plasma metabolomics, as it is a noninvasive, powerful, multivariate method to surveil the efficacy of gene therapy.

In addition, untreated *myd* mice displayed a significant decline in BW that progressed until euthanasia was required, whereas systemic gene transfer of *Large1* resulted in a mild gain in BW. Perhaps the most remarkable was the ability of AAV*Large1* to significantly extend the survival of older *myd* mice. Notably, all but one mouse injected with AAV*Large1* lived to >65 weeks of age, whereas only 50% of untreated *myd* mice survived to 45 weeks of age. Thus, results from our noninvasive assays provide evidence that α-DG function is restored following systemic treatment with AAV*Large1*, and that this has a significant impact on survival.

To further support our findings from noninvasive assays, we performed invasive analysis of muscle following euthanasia of mice treated with AAV*Large1*. Western blotting analysis and quantitative solid-phase laminin binding assays demonstrated that AAV*Large1* was able to fully restore matriglycan on α-DG. Systemic transduction with AAV*Large1* in *myd* mice resulted in a significant increase in *F*_o_ and *F*_o_/CSA of EDL muscles compared to untreated mice, demonstrating that muscle contractile properties can be improved, even at advanced stages of disease. In addition, EDL muscles in AAV*Large1*-treated mice tolerated a sequence of ECCs in a manner that was largely similar to that of WT mice. Collectively, our histological, biochemical, and physiological analysis of muscle shows that matriglycan is restored on α-DG, and muscle function improved in *myd* mice treated with AAV*Large1*. These results are in contrast to preclinical studies of Duchenne muscular dystrophy gene therapy, which have been hampered by the large size of the dystrophin complementary DNA (cDNA), and attempts to circumvent this by AAV-mediated gene transfer of microdystrophin did not significantly protect hindlimb muscles from contraction-induced injury (*8*, *15*).

The prospects for gene therapy for dystroglycanopathies are encouraging since all causative genes are less than 2.7 kb, well within the limits of AAV vectors (*25*). Dystroglycanopathies often involve the central nervous system (CNS), and *myd* mice exhibit CNS defects as well as muscular dystrophy (*31*). Thus, impaired CNS function in *myd* mice is a potential target for postnatal AAV*Large1* gene therapy. The potential of CNS gene therapy was shown in a recent study where postnatal AAV gene therapy improved spatial learning despite the presence of neural ectopia in a mouse model of neuronal migration disorder (*38*). In our study, RO injection of AAV*Large1* only slightly increased *Large1* expression in aged *myd* brain and thus did not restore matriglycan or α-DG ligand binding. Further investigation is necessary to test whether CNS-targeted *Large1* gene therapy in the *myd* mouse can restore CNS function.

Individuals with muscular dystrophy are commonly diagnosed after the onset of clinical symptoms, yet current treatments are unable to restore the function of degenerated muscles. We show that AAV*Large1* gene transfer in older *myd* mice with severe muscular dystrophy attenuates skeletal muscle degeneration, improves motor and respiratory function, and normalizes systemic metabolism, which collectively extends survival of *myd* mice. Furthermore, our results demonstrate that skeletal muscle has remarkable plasticity, suggesting that gene therapy that is aimed at rescuing deficits in gene function harbors the potential to effectively restore muscle function. Thus, this study provides proof of concept that functional and pathological features of muscular dystrophy are reversible, even in advanced stages of disease.

## MATERIALS AND METHODS

### Experimental design

We set out to evaluate the therapeutic effect of treating established α-dystroglycanopathy; therefore, we targeted aged, severely affected *Large^myd^*/*Large^myd^* (*myd*) mice as they exhibit end-stage muscle pathophysiology. The *myd* mouse is a natural model of glycosylation-deficient muscular dystrophy, with 65% of animals dying before 40 weeks of age (*29*–*31*). Previous work involving *Large1* gene transfer with adenovirus used direct muscle injections into young *myd* mice and showed localized prevention of dystrophic pathology (*12*). Successful systemic gene transfer using 10^12^ to 10^13^ vector genome (vg) per mouse via intraperitoneal or intravenous injections has previously been established for serotype 9, especially in skeletal muscle and heart (*8*–*13*). Therefore, we choose to use the AAV2/9 vector with a CMV promoter to systemically administer *Large1* to *myd* mice. Thirty-five surviving *myd* mice that were 34 weeks or older were randomly assigned to untreated or treated groups. In the treated group, a total of 14 mice were injected once intraperitoneally (*n* = 3) or once intravenously (*n* = 11) with AAV2/9CMV*Large1* (AAV*Large1*) at a vector dose of 4.35 × 10^12^ vg per mouse. Twenty-one mice were left untreated. Therapeutic readouts were BW, survival, muscle and respiratory function, muscle physiology, histological evaluation, plasma metabolomics, and biochemical analysis of α-DG (see Fig. 1A for experimental outline). The mice were weighed weekly, and locomotor activity was assessed every 4 weeks. The respiratory function test was performed just before untreated mice were euthanized or when AAV-treated mice became 60 weeks old.

### Animal care and criterion for euthanasia

Mice were maintained in a barrier-free, specific pathogen–free grade facility and had access to normal chow and water ad libitum. All animals were manipulated in biosafety cabinets and change stations using aseptic procedures. The mice were maintained in a climate-controlled environment at 25°C on a 12/12-hour light/dark cycle. All animal protocols were approved by the University of Iowa Institutional Animal Care and Use Committee (IACUC). MYD/Le-*Os*+/+*Large^myd^*/J mice (The Jackson Laboratory, stock #000300) were maintained on a C57BL/6J (WT) background, and colony maintenance was carried out in the laboratory by mating +/*myd* males to +/*myd* females. *myd* mice and control littermate mice (*+*/+ or +/*myd* mice) were identified via polymerase chain reaction (PCR).

The genetic background of our *myd/myd* (MYD/Le-*Os+/+Large^myd^*/J) mouse line (The Jackson Laboratory, #000300) was tested at Transnetyx (Cordova, TN) with an array-based platform using more than 10,000 single-nucleotide polymorphism markers, which showed the line has a 92.5% C57BL/6 genetic background. Animal care, ethical usage, and procedures were approved and performed in accordance with the standards set for by the National Institutes of Health (NIH) and IACUC. Of the 161 offspring from heterozygous +/*myd* matings, only 18 (11.2%) were homozygotes (*myd*/*myd*). Theoretically, there is a 25% chance that a homozygote is produced, suggesting that some homozygotes could be embryonic lethal. Surviving *myd* mice older than 35 weeks of age required gruel feeding in the cage, as they were too weak to reach food pellets suspended overhead. Because of the progressive deterioration in motor and respiratory function with age, *myd* mice were euthanized due to an inability to feed, hindlimb paralysis, reduced body score, or respiratory distress, as dictated in our IACUC protocol.

### AAV vector production and AAV injection

The sequence encoding mouse *Large1* was synthesized (GenScript, Piscataway, NJ) and cloned into the AAV backbone under the transcriptional control of the ubiquitous CMV promoter. The AAV2/9 vector contains the genome of serotype 2 packaged in the capsid from serotype 9 and was selected because of its ability to improve muscle transduction efficiency as well as alter tropism. The vector AAV2/9CMV*Large1* was generated by the University of Iowa Viral Vector Core Facility. For adult mice, 100 μl (4.35 × 10^12^ vg) of the vector solution was administered once intraperitoneally or intravenously via the RO sinus.

### Locomotor activity

Locomotor activity was measured using an open-field DigiScan apparatus (Omnitech Electronics, Columbus, OH). The mice were acclimated to the open-field apparatus for 3 days before the first trial. Total walking distance and rearing behavior (vertical activity) were recorded every 10 min for 1 hour when the mice were in active phase. Activity data were collected for two consecutive days, and the higher of the two values for each parameter was used for analysis.

### Forelimb grip strength test

The forelimb grip strength test was performed when mice were 60 weeks old. A mouse grip strength meter (Columbus Instruments, Columbus, OH) was mounted horizontally, with a nonflexible grid connected to the force transducer, which shows the highest force applied by the mouse on the grid during the pull. The mouse was allowed to grasp the grid with its front paws and then pulled away from the grid so that its grasp was broken. The gram force was recorded per pull, but measures were rejected in which only one forepaw or the hindlimbs were used, and in which the mouse turned during the pull. A total of 15 pulls (five series of three pulls in a row) were performed, with a resting period between series to allow the mouse to recover and avoid habit formation. The three highest values out of the 15 values collected were used to determine the maximum grip strength.

### Plethysmography

Respiratory function was tested in unrestrained mice using a whole-body plethysmograph (Buxco Respiratory Products, Data Sciences International, St. Paul, MN). The mouse was weighed and placed into the chamber for 15 min to acclimate, and respiratory flow data were recorded for 5 min using FinePointe software (Data Sciences International, St. Paul, MN). The measurements were done for two consecutive days. For data analysis, average values for TV, MV, TV/BW, and MV/BW were used.

### Measurement of in vitro muscle function

To compare the contractile properties of muscles, EDL muscles were surgically removed and analyzed as described previously (*39*, *40*), with minor modifications. The muscle was immediately placed in a bath containing a buffered physiological salt solution (137 mM NaCl, 5 mM KCl, 2 mM CaCl_2_, 1 mM MgSO_4_, 1 mM NaH_2_PO_4_, 24 mM NaHCO_3_, 11 mM glucose). The bath was maintained at 25°C, and the solution was bubbled with 95% O_2_ and 5% CO_2_ to stabilize the pH at 7.4. The proximal tendon was clamped to a post, and the distal tendon was tied to a dual-mode servomotor (model 305C; Aurora Scientific, Aurora, ON, Canada). Optimal current and whole muscle length (*L*_o_) were determined by monitoring isometric twitch force. Optimal frequency and maximal isometric tetanic force (*F*_o_) were also determined. The muscle was then subjected to an ECC protocol consisting of five ECCs separated by 3-min intervals. A fiber length (*L*_f_)–to–*L*_o_ ratio of 0.45 was used to calculate *L*_f_. Each ECC consisted of an initial 100-ms isometric contraction at optimal frequency immediately followed by a stretch of *L*_o_ to 30% of *L*_f_ beyond *L*_o_ at a velocity of one *L*_f_/s at optimal frequency. The muscle was then passively returned to *L*_o_ at the same velocity. At 3, 15, 30, 45, and 60 min after the ECC protocol, isometric tetanic force was measured, and force deficit was calculated as the decrease in isometric tetanic force after ECC as a percentage of *F*_o_. After the analysis of the contractile properties, the muscle was weighed. The CSA of muscle was determined by dividing the muscle mass by the product of *L*_f_ and the density of mammalian skeletal muscle (1.06 g/cm^3^). The specific force was determined by dividing *F*_o_ by the CSA (mN/mm^2^).

### Tissue collection and histological evaluation

Mice were euthanized by cervical dislocation, and tissue samples were obtained. Quadriceps, gastrocnemius, tibialis anterior, and soleus muscles were weighed before processing. Quadriceps and gastrocnemius muscles from the right side of the animal, half the diaphragm, and half the heart were snap-frozen in optimal cutting media (OCT), submerged in isopentane, cooled in liquid nitrogen, and stored at −80°C for further analysis. The other skeletal muscles were frozen in liquid nitrogen and stored at −80°C for biochemical analysis. Cryosections of quadriceps muscle, diaphragm, and heart were cut at a thickness of 7 μm and stained with hematoxylin and eosin (H&E) and Sirius red and Fast Green. For Sirius red staining, sections were fixed in 10% neutral buffered formalin (Thermo Fisher Scientific) for 5 min, followed by Sirius red staining consisting of a 30-min incubation in 0.1% Sirius red F3B (Chroma Gesellschaft, Germany, 1A 280) in saturated picric acid (Sigma-Aldrich, St. Louis, MO), and several incubations with ethyl alcohol and xylene. Whole digital images of H&E- and Sirius red–stained sections were taken by a VS120-S5-FL Olympus slide scanner microscope (Olympus Corporation, Tokyo, Japan). To quantify fibrous tissue infiltration in Sirius red–stained muscle and diaphragm sections, four fields were randomly selected from a whole section using OlyVIA ver.2.9 (Olympus) and the captured images were processed using ImageJ (NIH) with additional threshold color plug-ins to process jpeg images. Pixels corresponding to the area stained in red were normalized to the total pixel area of the image, and the four values were expressed as a ratio of the fibrotic area and were averaged for each section.

### Immunohistochemistry

For morphometric analyses, transverse sections of the sarcolemma of muscle, diaphragm, and heart were stained with a rabbit polyclonal anti–caveolin 3 antibody (1:100 dilution; Abcam, ab2912) and a mouse monoclonal antibody to glycoepitopes on the sugar chain of α-DG (1:100 dilution; VIA41) overnight at 4°C, followed by staining with Alexa Fluor–conjugated goat immunoglobulin G (IgG) against rabbit IgG and goat IgG against mouse IgG1 (1:400 dilution; Invitrogen), respectively, for 40 min. The sections were counterstained with 4′,6-diamidino-2-phenylindole (DAPI) (Invitrogen), and whole sections were imaged with a VS120-S5-FL Olympus slide scanner microscope. Feret’s diameter of muscle fibers was measured with VS-DESKTOP software (Olympus). To quantify fibers expressing glycosylated α-DG, the percent of VIA41-positive fibers was determined by dividing the VIA41-positive fiber count by the anti–caveolin 3–positive fiber count. To quantify fibers with centrally located nuclei, three fields were randomly selected from a whole section and were double-stained with anti–caveolin 3 antibody and DAPI. Total and centrally nucleated fibers (CNFs) were manually counted, and %CNF was determined by dividing CNF by total fiber count per each captured image. The three %CNF values were averaged and compared. The sarcolemma of muscle and diaphragm sections was also stained with a rabbit monoclonal antibody to β-DG (1:25 dilution, AP83) and VIA41. IIH6 and VIA41 antibodies are monoclonal antibodies to the glycoepitope of α-DG (*21*, *22*), and AP83 is a polyclonal antibody to the C terminus of β-DG (*20*), all of which have been described previously.

### Glycoprotein enrichment and Western blot analysis

Half of the quadriceps muscle was solubilized in 1 ml of tris-buffered saline (TBS) containing 1% Triton X-100 and protease inhibitors. The solubilized fraction was incubated with 200 μl of WGA–agarose bead slurry (Vector Laboratories, Burlingame, CA) overnight at 4°C. Pellets formed from the beads and were washed three times in 1 ml of TBS containing 0.1% Triton X-100 (*15*). The beads were then either directly mixed with SDS–polyacrylamide gel electrophoresis (PAGE) loading buffer (for Western blotting, ligand overlay) or eluted with 1 ml of TBS containing 0.1% Triton X-100 and 300 mM *N*-acetylglucosamine (for solid-phase binding assay). Proteins were separated by 3 to 15% SDS-PAGE and transferred to polyvinylidene difluoride–FL (PVDF-FL; Millipore Sigma) membranes. The membranes were incubated with a sheep polyclonal antibody to human DG (1:100 dilution; R&D Systems, AF6868) and a mouse monoclonal antibody to a glycoepitope on the sugar chain of α-DG (IIH6; 1:100 dilution) followed by IRDye 800CW dye-conjugated goat anti-sheep IgG (LI-COR, 926-32214) and goat anti-mouse IgM (LI-COR, 926-32280), respectively.

### Ligand overlay assay

Ligand overlay assays were performed on PVDF-FL membranes using mouse Engelbreth-Holm-Swarm (EHS) laminin (Thermo Fisher Scientific, 23017015). Briefly, PVDF-FL membranes were blocked in laminin binding buffer [LBB; 10 mM triethanolamine, 140 mM NaCl, 1 mM MgCl_2_, 1 mM CaCl_2_ (pH 7.6)] containing 5% milk followed by incubation with laminin overnight at 4°C in LBB containing 3% bovine serum albumin (BSA). Membranes were washed and incubated with anti-laminin antibody (1:100 dilution; Sigma-Aldrich, L9393) followed by IRDye 800CW dye-conjugated donkey anti-rabbit IgG (LI-COR, 926-32213).

### Solid-phase assay

A solid-phase laminin binding assay was performed as described previously (*17*). Briefly, WGA eluates were diluted 1:50 in TBS and coated on polystyrene enzyme-linked immunosorbent assay microplates (Costar 3590) overnight at 4°C. Plates were washed in LBB and blocked for 2 hours in 3% BSA/LBB at room temperature (RT). Mouse EHS laminin was diluted in 1% BSA/LBB and applied for 1 hour at RT. The wells were washed with 1% BSA/LBB and incubated for 1 hour with L9393 (1:5000 dilution) in 3% BSA/LBB followed by incubation with horseradish peroxidase–conjugated anti-rabbit IgG (1:5000 dilution; Invitrogen) in 3% BSA/LBB for 30 min. Plates were developed with *o*-phenylenediamine dihydrochloride and H_2_O_2_, and reactions were stopped with 2 N H_2_SO_4_. Absorbance per well was read at 490 nm with a microplate reader.

### mRNA expression analysis

Quadriceps muscle and heart cryosections were collected into tubes and stored at −80°C until use. Total RNA was isolated using an RNeasy Mini kit (Qiagen, Venlo, The Netherlands), and cDNA was generated using the QuantiTect Reverse Transcription Kit (Qiagen). For gene expression analysis, a QX100 droplet digital PCR (ddPCR) system (Bio-Rad, Pleasanton, CA) was used. The ddPCR reaction mixture consisted of 10 μl of a 2× EvaGreen Supermix (Bio-Rad), 2 μl of primers, and 5 μl of cDNA sample in a final volume of 20 μl. The entire reaction mixture was loaded into a disposable plastic cartridge (Bio-Rad) together with 70 μl of droplet generation oil (Bio-Rad) and placed in the droplet generator (Bio-Rad). After processing, the droplets generated from each sample were transferred to a 96-well PCR plate (Eppendorf, Hamburg, Germany). PCR amplification was carried out on a T100 thermal cycler (Bio-Rad) using a thermal profile beginning at 95°C for 5 min, followed by 40 cycles of 95°C for 30 s and 60°C for 60 s, one cycle of 90°C for 5 min, and ending at 4°C. After amplification, the plate was loaded on the droplet reader (Bio-Rad) and the droplets from each well of the plate were read automatically at a rate of 32 wells per hour. ddPCR data were analyzed with QuantaSoft analysis software (Bio-Rad), and the quantification of the target molecule was presented as the number of copies per microliter of PCR mixture. The oligonucleotides used for amplification are listed in table S2.

### Targeted metabolomics

Forty microliters of plasma was vortexed with 720 μl of ice-cold 1:1 methanol/acetonitrile to extract metabolites and incubated for 1 hour at −20°C. Metabolite extracts were centrifuged for 10 min at 21,000*g* to pellet precipitated protein. Supernatants were transferred to sample vials and dried using SpeedVac. The resulting dried metabolite extracts were derivatized using methoxyamine hydrochloride (MOX) and *N*,*O*-bis(trimethylsilyl)trifluoroacetamide (TMS) and examined by gas chromatography–mass spectrometry (GC-MS), as previously described (*41*, *42*). Briefly, dried extracts were reconstituted in 30 μl of MOX (11.4 mg/ml) in anhydrous pyridine, vortexed for 5 min, and heated for 1 hour at 60°C. Next, 20 μl of TMS was added to each sample, which was then vortexed for 1 min and heated for 30 min at 60°C. Samples were immediately analyzed using GC-MS. GC separation was conducted on a Thermo Trace 1300 GC fitted with a TraceGold TG-5SilMS column. One microliter of derivatized sample was injected into the GC operating under the following conditions: split ratio = 20-1, split flow = 24 μl/min, purge flow = 5 ml/min, carrier mode = constant flow, and carrier flow rate = 1.2 ml/min. The GC oven temperature gradient was as follows: 80°C for 3 min, increasing at a rate of 20°C/min to 280°C, and holding at a temperature at 280°C for 8 min. Metabolites were detected using a Thermo ISQ 7000 mass spectrometer operated from 3.90 to 21.00 min in electron ionization (EI) mode (−70 eV) using select ion monitoring. EI fragmented metabolites were identified by the mass/charge ratio (*m/z*) of fragments at unique chromatographic retention times corresponding to previously analyzed reference standards. Peak intensities were corrected using the NOREVA tool (*43*). Peak intensities from six unique experiments were normalized to total signal per sample and set relative to WT for final analysis.

### Statistical analysis

All data in the present study are shown as the means ± SEM unless otherwise indicated. The number of sampled units, *n*, upon which we reported statistics, is the single mouse for the in vivo experiments (one mouse is *n* = 1). GraphPad Prism 7 software was used for statistical analyses. Mann-Whitney or unpaired *t* test was used between untreated and treated mice. Locomotor activity was compared between baseline (pre) and indicated weeks with Wilcoxon signed rank or paired *t* test. *P* < 0.05 was considered significant. For survival, treated mice were compared with untreated mice by Kaplan-Meier log-rank test. For all figures, *P < 0.05, **P < 0.01, ***P < 0.001, and ****P < 0.0001 were used.

## Notes

View/request a protocol for this paper from *Bio-protocol*.
