# Supplementary material for: Large1 gene transfer in older myd mice with severe muscular dystrophy restores muscle function and greatly improves survival
Source: Sci Adv. 2022 May 25;8(21):eabn0379. doi: 10.1126/sciadv.abn0379 (PMC9132445; doi:10.1126/sciadv.abn0379)
Supplement: Supplementary file 1 — Figs. S1 to S7 Tables S1 and S2 [file sciadv.abn0379_sm.pdf]

Science Advances
AAAS

Supplementary Materials for

Large1 gene transfer in older *myd* mice with severe muscular dystrophy restores muscle function and greatly improves survival

Takahiro Yonekawa *et al.*

Corresponding author: Eric B. Taylor, eric-taylor@uiowa.edu; Kevin P. Campbell, kevin-campbell@uiowa.edu

Sci. Adv. **8**, eabn0379 (2022)
DOI: 10.1126/sciadv.abn0379

This PDF file includes:

Figs. S1 to S7
Tables S1 and S2

SUPPLEMENTAL FIGURES

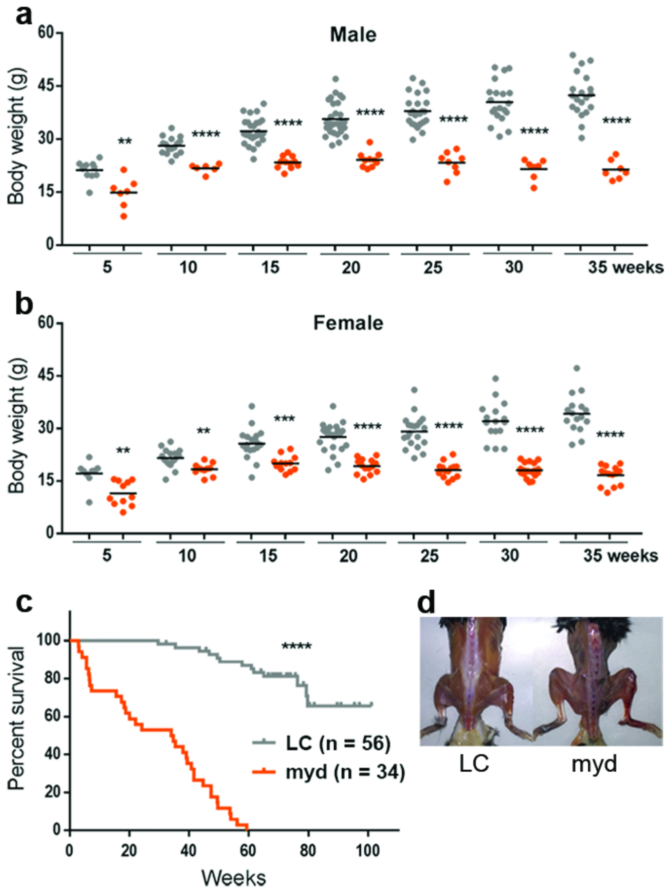

Fig. S1 │ *myd* mice show growth retardation, a shortened lifespan, and muscle wasting. **a, b,** Body weights of male (**a**; littermate control (LC): n=20, *myd*: n=7) and female (**b**; LC: n=16; *myd*: n=14) mice, which were weighed weekly, with weights compared at 5-week intervals. **c,** Percent survival of LC and *myd* mice. **d,** Image of a *myd* mouse depicting thoracic kyphosis and muscle wasting; 68.1-week-old LC mouse (left), 41.6-week-old *myd* mouse (right). For panels **a** and **b**, symbols represent individual mice, bars represent means. LC are gray symbols; *myd* (myd) are orange symbols. * $p<0.05$; ** $p<0.01$, *** $p<0.001$; **** $p<0.0001$.

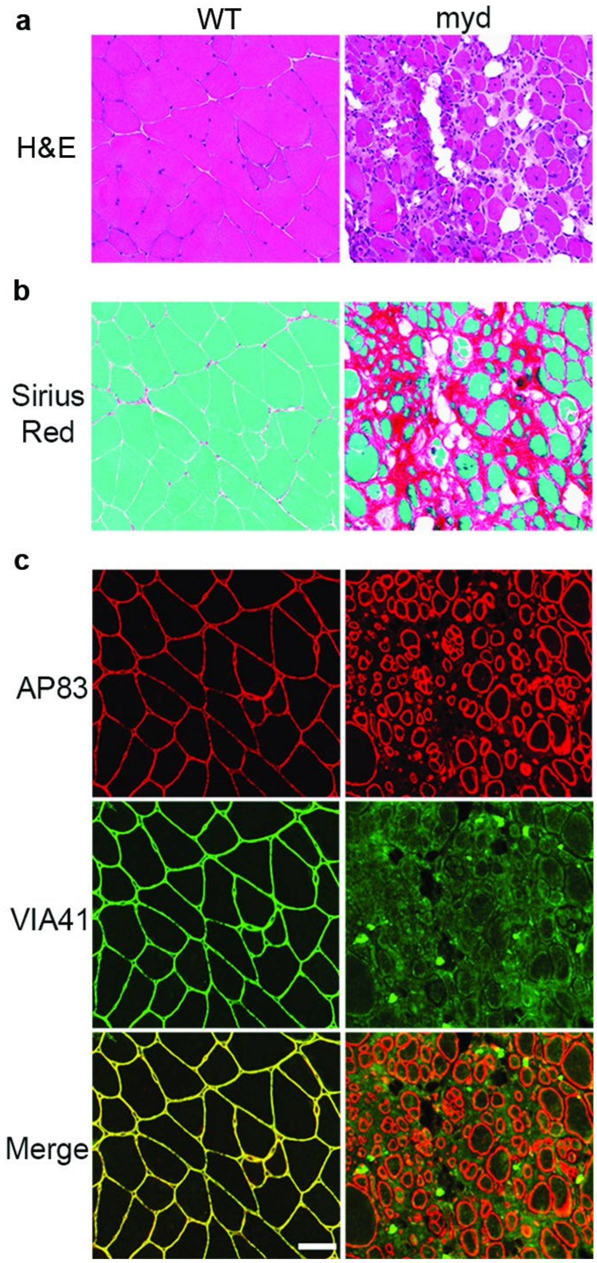

Fig. S2 │ Quadricep muscles from aged *myd* mice exhibit severe dystrophic pathology and lack matriglycan.

Quadricep muscle cryosections from a 69.0-week-old C57BL/6J (WT) mouse and a 41.6-week-old *myd* (myd) mouse. **a, b,** Cryosections stained with H&E or Sirius Red & Fast Green. **c,** Immunofluorescence analysis of cryosections for β-DG (AP83) or matriglycan (VIA41). Scale bar represents 50 μm.

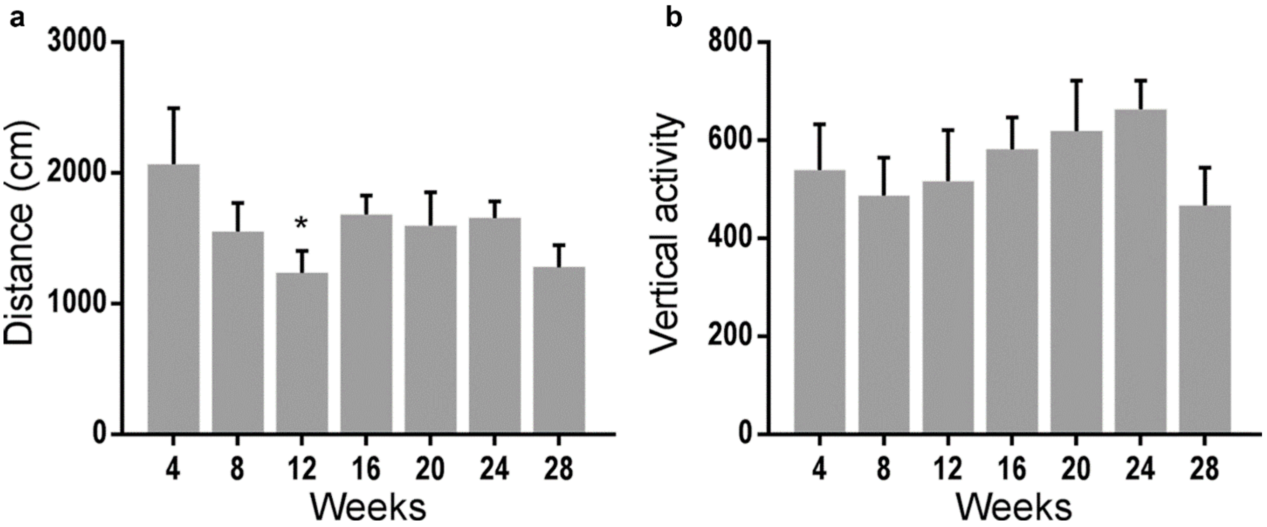

Fig. S3 │Chronological evaluation of locomotion and rearing activity in C57BL/6J WT mice.

a, b, Spontaneous locomotion (distance) and rearing (vertical activity) were measured every four weeks in C57Bl/6J WT mice. Paired t-tests were used to compare the parameters between baseline activity and that at 4, 8, 12, 16, 20, 24, and 28 weeks (n = 6). Bars represent mean ± SEM. * $p<0.05$.

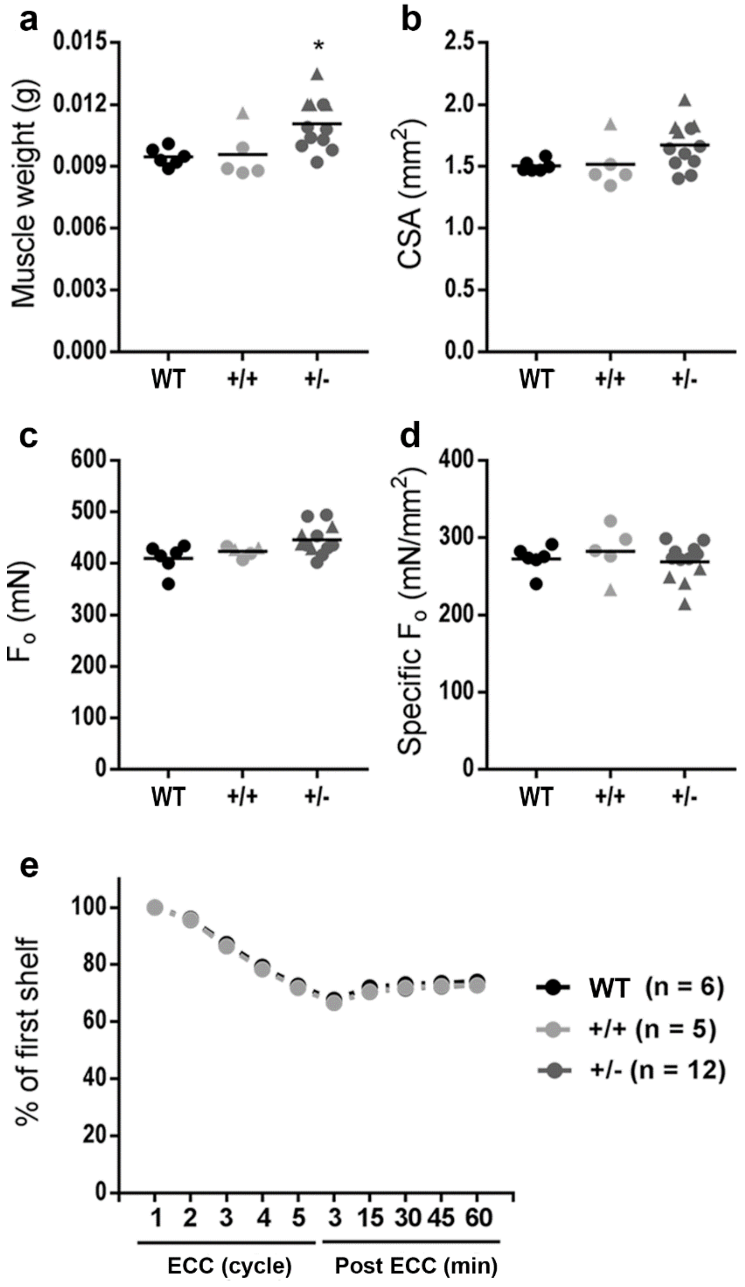

Fig. S4 │Muscle size, contractile properties, and lengthening contraction-induced muscle damage is similar in EDL muscles from +/+ or +/*myd* mice.

a, Muscle weight, **b,** whole-muscle CSA, **c,** isometric tetanic force (F_o), and **d,** specific isometric tetanic force (F_o/CSA) were measured in C57Bl/6J (WT), +/+, and +/*myd* (+/-) mice. Unpaired t-tests were used to compare the parameters between +/+ and +/*myd* mice. **e,** Percentages of F_o at the 2nd, 3rd, 4th, or 5th eccentric contraction (ECC) cycle, and at 3, 15, 30, 45, and 60 min after the eccentric contraction protocol (Post ECC) were calculated as a relative value to F_o at the 1st ECC, in mice as in (a). Filled triangles are male; filled circles are female. WT are black symbols; +/+ are light gray symbols; +/- are dark gray symbols. * $p<0.05$.

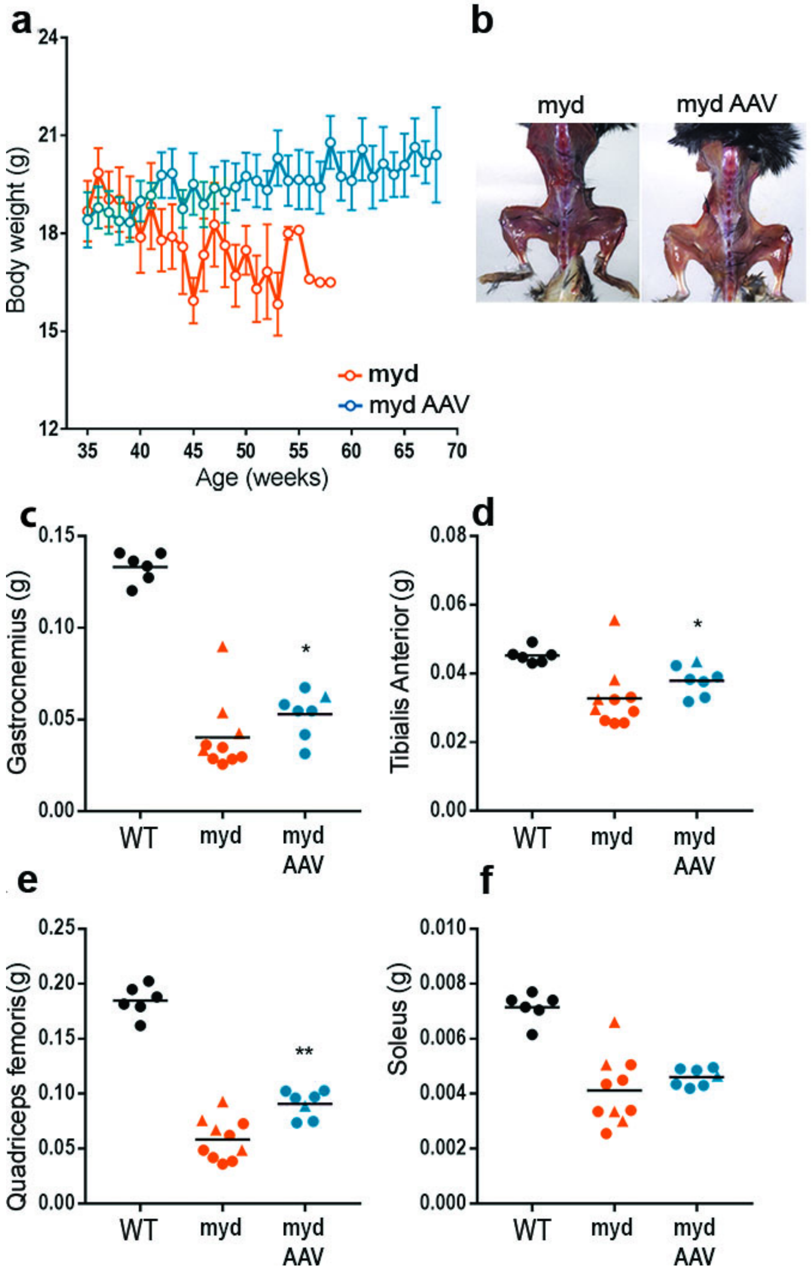

Fig. S5 │ AAV-mediated *Large1* gene transfer improves muscle mass in *myd* mice.
a, Body weight of untreated (orange) or AAV*Large1*-treated (blue) *myd* mice. Mice were treated with AAV*Large1* at >35 weeks and weighed weekly for at least 30 weeks. Linear regression analysis was performed, and both slopes were significantly non-zero: untreated, $Y = -0.1125*X + 22.88$; treated, $Y = 0.05238*X + 16.85$. **b,** Gross analysis of mice treated as in (**a**); 40.7-week-old untreated mouse (left), 68.1-week-old treated mouse (right). **c-f,** Weight of the gastrocnemius **c**, tibialis anterior **d**, quadriceps femoris **e**, and soleus **f,** muscles from C57Bl/6J (WT) mice, untreated (myd), or treated (myd AAV) *myd* mice as in (**a**). Average weights were compared between untreated and treated groups using Mann-Whitney or unpaired t-test. Symbols represent individual mice, bars represent mean ± SEM. Filled triangles are male; filled circles are female. WT are black symbols; myd are orange symbols; myd AAV are blue symbols. * $p<0.05$; ** $p<0.01$.

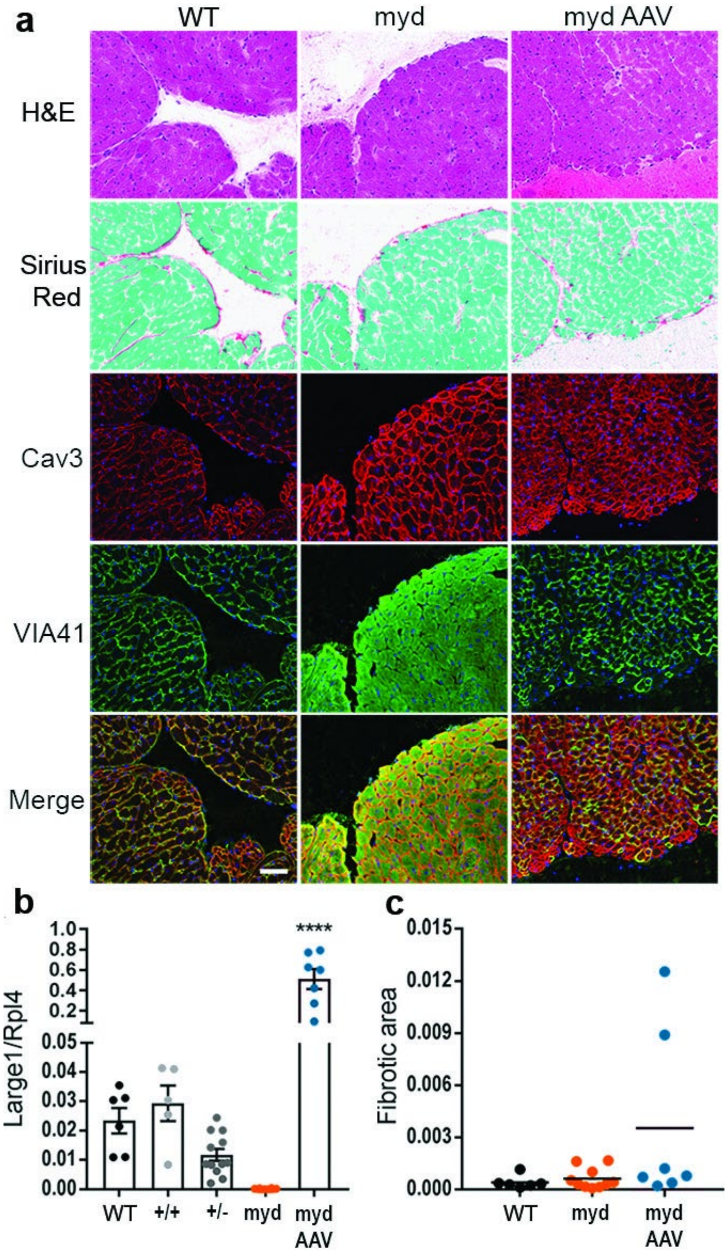

Fig. S6 │Restoring *Large1* expression improves muscle pathology, immunohistochemistry, and morphometric analysis of cardiac transverse sections.

a, Representative cardiac transverse sections from a 68.1-week-old C57BL/6J (WT) mouse, a 56.1-week-old *myd* (myd) mouse, and a 69.3-week-old *myd* mouse treated with AAV*Large1* (myd AAV). From top to bottom: sections stained with H&E, sections stained with Sirius Red & Fast Green, and immunofluorescence analysis of sections for: β-DG (AP83) and matriglycan (VIA41). Scale bar represents 50μm. **b,** ddPCR analysis of *Large1* expression relative to *Rpl4* expression in cardiac muscle from C57Bl/6J mice (WT; n = 6), +/+ (n = 5) and +/*myd* (+/-; n = 12), untreated *myd* (myd; n = 10), and *myd* mice treated with AAV*Large1* (myd AAV; n = 7). Symbols represent individual mice, bars represent mean ± SEM. **c,** Connective tissue deposition to assess fibrosis, determined by Sirius Red & Fast Green staining in (**a**). Untreated and treated groups compared with unpaired t-test.

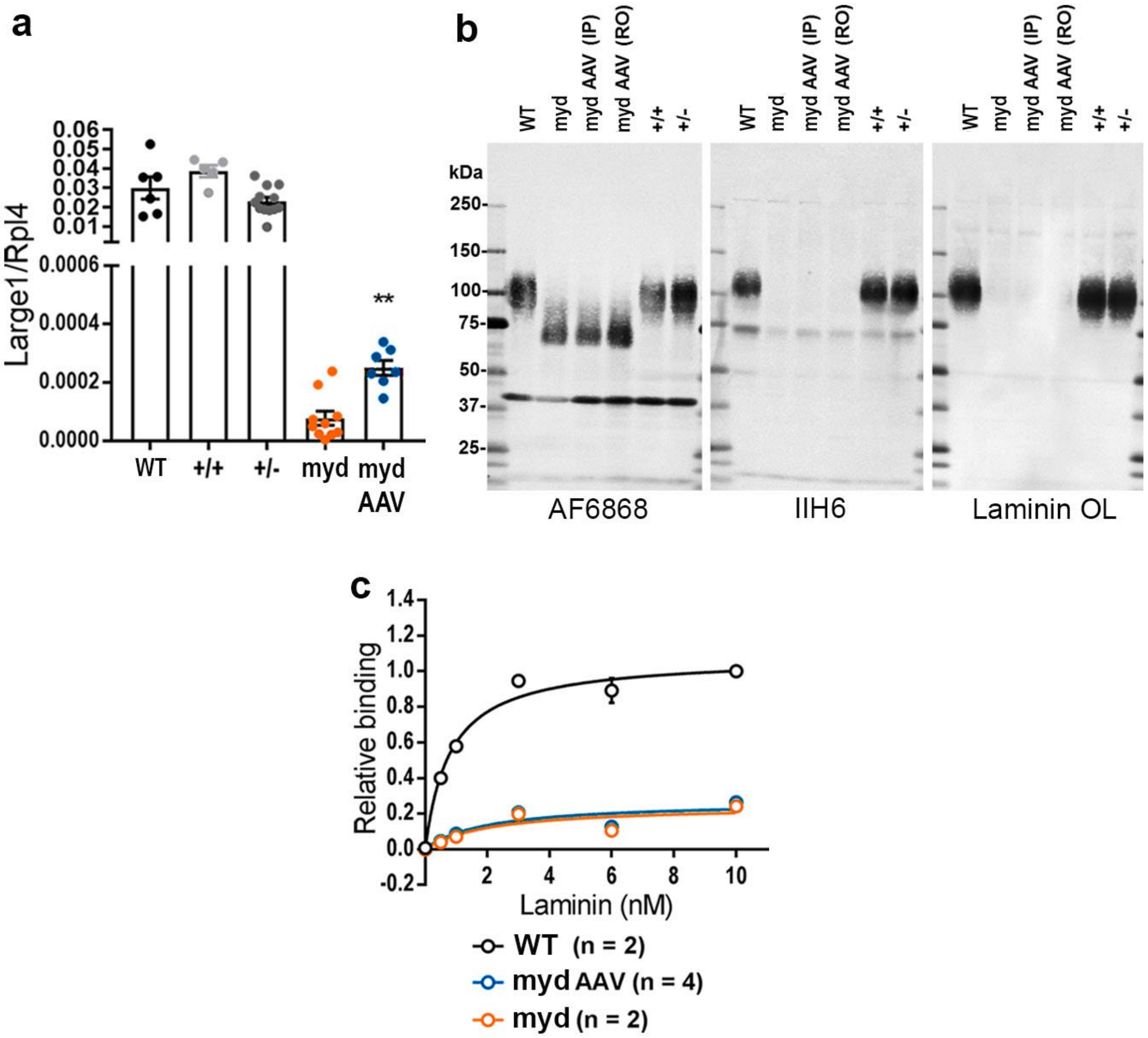

Fig. S7 │ *Large1* Expression, biochemical analysis of α-DG and α-DG–ligand binding in the brain of wild-type, *myd,* and *myd* mice injected with AAV*Large1*.

a, ddPCR analysis of *Large1* expression relative to *Rpl4* expression (*Large1/Rpl4*) in brains from aged C57Bl/6J mice (WT; n = 6), +/+ (n = 5), +/*myd* (+/-; n = 12), untreated *myd* mice (myd; n = 10), and *myd* mice treated with AAV*Large1* (myd AAV; n = 7). Symbols represent individual mice, bars represent mean ± SEM. Mann-Whitney test was used to compare gene expression between treated and untreated *myd* mice. WT are black symbols; +/+ are light gray symbols; +/- are dark gray symbols; myd are orange symbols; myd AAV are blue symbols. **b,** Western blot analysis of WGA-enriched brain homogenates from aged C57Bl/6J mice (WT), +/+, +/*myd* (+/-), untreated *myd* (myd), or *myd* mice treated with AAV*Large1* via intraperitoneal (myd AAV (IP)) or retro-orbital (myd AAV (RO)) injection to assess expression of β-DG (AF6868), α-DG reactive to IIH6, or laminin (laminin OL). **c,** Solid-phase binding assay of WGA-enriched brain glycoproteins of aged C57Bl/6J (WT), untreated *myd* (myd), or *myd* mice treated with AAV*Large1* (myd AAV). **p<0.01.

SUPPLEMENTARY DATA TABLES

Metabolite	+/+	Myd	Myd AAV	p-values			
				One-Way ANOVA	Post-hoc (Tukey's multiple comparison test)		
					+/+ vs Myd	+/+ vs Myd AAV	Myd vs Myd AAV
1-Octadecanol	1.00 ± 0.022	0.96 ± 0.027	0.99 ± 0.029	0.587	0.5841	0.9829	0.7105
2-Hydroxybutyrate	1.00 ± 0.05	1.07 ± 0.069	0.89 ± 0.09	0.2164	0.7547	0.4998	0.1953
2-Hydroxyglutarate	1.00 ± 0.079	0.37 ± 0.03	0.71 ± 0.112	<0.0001	<0.0001	0.0351	0.022
2-Oxoadipate	1.00 ± 0.051	0.97 ± 0.053	1.25 ± 0.132	0.0496	0.9705	0.0915	0.0749
3-Phosphoglycerate	1.00 ± 0.043	1.49 ± 0.116	1.12 ± 0.092	0.0004	0.0003	0.5389	0.0107
6-Phosphogluconate	1.00 ± 0.055	0.96 ± 0.057	0.85 ± 0.059	0.1644	0.8472	0.1462	0.4382
Aconitate	1.00 ± 0.12	0.88 ± 0.115	1.15 ± 0.152	0.3796	0.7913	0.6927	0.3487
Adonitol	1.00 ± 0.055	0.9 ± 0.053	1.13 ± 0.054	0.0219	0.4288	0.2011	0.0169
Alanine	1.00 ± 0.046	0.94 ± 0.072	1.19 ± 0.088	0.0348	0.8232	0.1076	0.0393
alpha-Keto-beta-Methylvalerate (KMV)	1.00 ± 0.053	0.67 ± 0.034	0.71 ± 0.099	0.0011	0.0025	0.0079	0.8941
alpha-Ketoglutarate	1.00 ± 0.061	0.52 ± 0.039	0.87 ± 0.087	<0.0001	<0.0001	0.3375	0.0019
alpha-Ketoisocaproate (KIC)	1.00 ± 0.034	0.76 ± 0.032	0.86 ± 0.047	0.0002	0.0001	0.0251	0.2131
alpha-Ketoisovalerate (KIV)	1.00 ± 0.059	0.77 ± 0.057	0.8 ± 0.09	0.0424	0.0594	0.1124	0.9413
Aminoadipate	1.00 ± 0.036	0.9 ± 0.048	1.07 ± 0.047	0.0418	0.2694	0.4913	0.0325
Arachidic acid (Eicosanoic acid)	1.00 ± 0.024	0.98 ± 0.037	0.89 ± 0.037	0.0375	0.9019	0.0379	0.1359
Asparagine	1.00 ± 0.055	1.2 ± 0.072	1.39 ± 0.095	0.0013	0.1402	0.0009	0.2001
Aspartate	1.00 ± 0.043	1.00 ± 0.074	0.85 ± 0.053	0.0973	>0.9999	0.1287	0.1635
beta-Alanine	1.00 ± 0.018	0.97 ± 0.029	0.97 ± 0.026	0.6082	0.7267	0.6295	0.991
beta-Hydroxy beta-Methylbutyric acid (HMB)	1.00 ± 0.018	1.02 ± 0.031	1.00 ± 0.03	0.7862	0.7949	0.9979	0.839
Cholesterol	1.00 ± 0.029	0.94 ± 0.034	0.78 ± 0.043	0.0001	0.4871	<0.0001	0.008
Citraconate	1.00 ± 0.063	0.87 ± 0.086	0.98 ± 0.097	0.5211	0.5203	0.9816	0.6531
Citrate	1.00 ± 0.038	0.81 ± 0.04	0.79 ± 0.061	0.0029	0.0172	0.0056	0.9503
Citrulline	1.00 ± 0.044	1.08 ± 0.054	1.15 ± 0.061	0.0786	0.232	0.7666	0.0717
Cysteine	1.00 ± 0.043	0.86 ± 0.058	1.06 ± 0.074	0.5211	0.5203	0.9816	0.6531
Dihydroxyacetone phosphate (DHAP)	1.00 ± 0.118	2.14 ± 0.287	1.14 ± 0.155	0.0001	0.0002	0.8511	0.0012
Fructose	1.00 ± 0.042	1.2 ± 0.141	1.19 ± 0.109	0.2608	0.3354	0.3497	0.9979
Fructose 6-phosphate	1.00 ± 0.05	1.47 ± 0.131	0.95 ± 0.068	<0.0001	0.0006	0.9071	0.0002
Fumarate	1.00 ± 0.044	0.72 ± 0.054	0.87 ± 0.068	0.0035	0.0023	0.1929	0.2001
Gamma-aminobutyric acid (GABA)	1.00 ± 0.085	1.14 ± 0.079	1.17 ± 0.131	0.3893	0.5641	0.4166	0.9764
Glucose	1.00 ± 0.023	0.81 ± 0.049	0.82 ± 0.034	0.0002	0.0011	0.0014	0.9854
Glucose 6-phosphate	1.00 ± 0.125	1.56 ± 0.159	1.15 ± 0.292	0.0042	0.017	0.8515	0.006
Glutamate	1.00 ± 0.03	0.95 ± 0.042	0.89 ± 0.038	0.0815	0.6009	0.0654	0.4514
Glutamine	1.00 ± 0.041	1.11 ± 0.052	1.01 ± 0.063	0.3046	0.3128	0.9794	0.4392
Glycerate	1.00 ± 0.02	1.09 ± 0.04	1.09 ± 0.041	0.09	0.1751	0.1242	0.992
Glycerol	1.00 ± 0.064	0.8 ± 0.058	0.92 ± 0.119	0.2086	0.1827	0.7675	0.5755
Glycerol Monolaurate (GML)	1.00 ± 0.046	0.89 ± 0.039	0.8 ± 0.054	0.0124	0.2426	0.0091	0.4016
Glycine	1.00 ± 0.04	1.12 ± 0.032	1.16 ± 0.068	0.047	0.1981	0.0472	0.8227
Heneicosylic acid (Heneicosanoic acid)	1.00 ± 0.02	0.96 ± 0.03	1.03 ± 0.053	0.0011	0.1231	0.0957	0.0007
Heptadecanoic acid	1.00 ± 0.018	1.08 ± 0.029	0.92 ± 0.035	0.3855	0.7115	0.7797	0.3519
Histidine	1.00 ± 0.04	1.17 ± 0.052	1.14 ± 0.056	0.0359	0.0454	0.1179	0.8885
Hypoxanthine	1.00 ± 0.043	1.27 ± 0.146	1.06 ± 0.036	0.0715	0.0644	0.8363	0.2249
Inositol	1.00 ± 0.049	1.37 ± 0.116	1.25 ± 0.074	0.0042	0.004	0.0634	0.543
Isocitrate	1.00 ± 0.048	0.88 ± 0.05	0.78 ± 0.055	0.0117	0.2154	0.0088	0.4321
Isoleucine	1.00 ± 0.045	1.04 ± 0.048	1.17 ± 0.06	0.0562	0.8237	0.0503	0.2228
Lactate	1.00 ± 0.024	0.69 ± 0.038	0.86 ± 0.041	<0.0001	<0.0001	0.0142	0.0023
Lauric acid	1.00 ± 0.039	1.01 ± 0.036	0.95 ± 0.042	0.443	0.9614	0.5767	0.4579
Leucine	1.00 ± 0.068	1.1 ± 0.063	1.37 ± 0.119	0.0084	0.7004	0.0071	0.0846
Linoleate	1.00 ± 0.029	1.23 ± 0.066	1.01 ± 0.057	0.0029	0.0053	0.9949	0.0097
Linolenic acid	1.00 ± 0.057	1.48 ± 0.12	1.1 ± 0.108	0.0017	0.0016	0.7285	0.0189
Lysine	1.00 ± 0.039	1.35 ± 0.067	1.23 ± 0.061	<0.0001	<0.0001	0.0082	0.294
Malate	1.00 ± 0.044	0.62 ± 0.05	0.86 ± 0.066	<0.0001	<0.0001	0.1551	0.0089
Mannose	1.00 ± 0.055	0.99 ± 0.072	0.75 ± 0.059	0.0068	0.9966	0.0114	0.0229
Methionine	1.00 ± 0.067	1.18 ± 0.098	1.38 ± 0.131	0.0263	0.4049	0.0195	0.3693
Myristic acid	1.00 ± 0.067	1.1 ± 0.077	0.91 ± 0.099	0.2949	0.6603	0.7116	0.2625
N-acetylglutamate	1.00 ± 0.052	0.86 ± 0.036	0.86 ± 0.022	0.0211	0.0474	0.0407	0.9851
Oleic acid	1.00 ± 0.047	1.23 ± 0.078	0.94 ± 0.078	0.0116	0.0504	0.7757	0.0123
O-Phosphoethanolamine	1.00 ± 0.048	1.16 ± 0.072	1.00 ± 0.053	0.0948	0.1268	>0.9999	0.1443
Ornithine	1.00 ± 0.053	1.24 ± 0.11	1.26 ± 0.069	0.0273	0.0781	0.0421	0.9792
Orotate	1.00 ± 0.056	1.15 ± 0.192	0.85 ± 0.064	0.1897	0.5754	0.5797	0.1628
Palmitate	1.00 ± 0.026	1.11 ± 0.037	0.92 ± 0.048	0.0023	0.0799	0.2368	0.0015
Phenylalanine	1.00 ± 0.04	1.11 ± 0.052	1.17 ± 0.053	0.0303	0.2109	0.0266	0.6754
Phosphoenolpyruvate	1.00 ± 0.039	1.02 ± 0.039	0.93 ± 0.038	0.2564	0.9362	0.3976	0.2718
Proline	1.00 ± 0.079	1.15 ± 0.122	1.49 ± 0.145	0.0102	0.6369	0.0081	0.1141
Pyruvate	1.00 ± 0.046	0.74 ± 0.082	0.82 ± 0.049	0.0077	0.0079	0.0765	0.6341
Ribose	1.00 ± 0.055	0.84 ± 0.065	0.8 ± 0.043	0.0265	0.1088	0.0317	0.894
Serine	1.00 ± 0.048	1.23 ± 0.068	1.38 ± 0.087	0.0006	0.0519	0.0005	0.3112
Stearate	1.00 ± 0.024	1.03 ± 0.03	0.91 ± 0.039	0.0225	0.7563	0.0899	0.0241
Succinate	1.00 ± 0.141	0.99 ± 0.206	0.67 ± 0.044	0.1868	0.998	0.2182	0.2867
Taurine	1.00 ± 0.075	1.34 ± 0.106	1.08 ± 0.11	0.0406	0.0352	0.8266	0.1642
Threonine	1.00 ± 0.048	1.18 ± 0.074	1.28 ± 0.094	0.0195	0.1871	0.0165	0.6078
Thymine	1.00 ± 0.064	1.00 ± 0.049	1.21 ± 0.101	0.08	0.9993	0.112	0.1352
Tryptophan	1.00 ± 0.037	1.06 ± 0.06	1.15 ± 0.042	0.0696	0.5937	0.0553	0.4184
Tyrosine	1.00 ± 0.069	1.12 ± 0.107	1.4 ± 0.143	0.0281	0.6931	0.0229	0.1929
Uracil	1.00 ± 0.088	0.98 ± 0.051	0.98 ± 0.072	0.9736	0.9727	0.9874	0.997
Urea	1.00 ± 0.03	0.98 ± 0.061	1.03 ± 0.037	0.7256	0.9267	0.8852	0.7047
Valine	1.00 ± 0.043	1.00 ± 0.047	1.14 ± 0.062	0.0978	0.9984	0.1356	0.1554
Xanthine	1.00 ± 0.206	2.64 ± 0.828	1.87 ± 1.053	0.1688	0.1476	0.6242	0.7203
Xylose	1.00 ± 0.124	0.99 ± 0.174	0.66 ± 0.059	0.111	0.9974	0.1361	0.1944

Data Table 1 │Plasma Metabolomics

Relative fold change of plasma metabolites in WT (+/+, n = 26), *myd* (myd, n = 20), and *myd* mice treated with AAV*Large1* (myd AAV, n = 22). Data are presented as mean ± SEM. Analysis by one-way ANOVA followed by Tukey's post hoc multiple comparison test. p-values from statistical analysis are reported.

Data Table 2 │Primer design

Gene	Forward	Reverse	Amplicon size (bp)
Large1	acc tgc agt gcg agt aga c	cct gtt gcc ctt tga act tat gg	224
Rpl4	cca aga cta tgc gca gga at	tgt ctg cag tcc cct tct ct	135
